# Epidemiology of overweight in under-five children in India: insights from National Family Health Survey

**DOI:** 10.1017/S0007114524001582

**Published:** 2024-09-14

**Authors:** Rukman Manapurath, Ranadip Chowdhury, Sunita Taneja, Nita Bhandari, Tor A. Strand

**Affiliations:** 1 Centre for International Health, University of Bergen, Bergen, Norway; 2 Nutrition, Society for Applied Studies, New Delhi, India; 3 Department of Research, Innlandet Hospital Trust, Lillehammer, Norway

**Keywords:** Paediatric obesity, Health surveys, Survey data analysis, Under-five children, India

## Abstract

Childhood overweight is not only an immediate health concern due to its implications but also significantly increases the risk of persistent obesity and consequently CVD in the future, posing a serious threat to public health. The objective of this study was to examine the trends and associated factors of childhood overweight in India, using nationally representative data from three rounds of the National Family Health Survey (NFHS). For the primary analysis, we used data from 199 375 children aged 0–59 months from fifth round of the NFHS (NFHS-5). Overweight was defined as BMI-for-age Z (BMI Z) score > +2 sd above the WHO growth standards median. We compared the prevalence estimates of childhood overweight with third round of the third round of NFHS and fourth round of the NFHS. Potential risk factors were identified through multiple logistic regression analyses. The prevalence of overweight increased from 1·9 % in third round of NFHS to 4·0 % in NFHS-5, a trend seen across most states and union territories, with the Northeast region showing the highest prevalence. The BMI Z-score distributions from the latest two surveys indicated that the increase in overweight was substantially larger than the decrease in underweight. The consistent upward trend in the prevalence across different demographic groups raises important public health concerns. While undernutrition rates have remained relatively stable, there has been a noticeable rise in the incidence of overweight during the same time frame. The increasing trend of overweight among children in India calls for immediate action.

Childhood overweight is a growing public health concern^(1)^. In 2020, it was estimated that 5·7 percent, or 38·9 million children under five worldwide, were overweight^(2)^. Reflecting this global concern, India’s most recent fifth round of National Family Health Survey (NFHS-5), a large-scale, nationally representative survey conducted at varying intervals over the past two decades, has highlighted this issue^(2)^. The NFHS-5 data indicate a 60 % increase in the prevalence of childhood overweight (defined by weight-for-height > 2 sd) compared with the fourth round of NFHS (NFHS-4)^(3)^. Notably, this trend poses a significant challenge to the achievement of Sustainable Development Goal 2·2, which aims to end all forms of malnutrition, including childhood overweight, by the year 2030^(4)^.

Overweight is usually defined by the BMI-for-age Z (BMI Z) score, a standardised measure for age- and sex-specific variations in children’s BMI. Although there are more accurate markers of adiposity, the Z score remains valuable at a population level due to its ability to measure deviation from the population average BMI^(5)^. Nearly 90 % of children who were obese (BMI Z > 3 sd) at age 3 remained overweight or obese during adolescence, with the highest increase in annual BMI increments occurring between ages 2 and 6^(6)^.

Excess body weight or obesity in childhood is associated with increased cholesterol, TAG and lower HDL-cholesterol and subsequently developing atherosclerosis in later childhood and adolescence^(7–9)^. Furthermore, these conditions are indicative of poor cardiometabolic health, evidenced by elevated metabolic syndrome risk scores at ages 10–11^(10)^. Interventions that caused even a modest reduction in BMI Z-score in obese children have been associated with improvements in several cardiovascular risk factors^(11)^.

While various initiatives and policies have focussed on school-age children, such as restricting trans fatty acids in food products and promoting the Fit India Movement, the under-five age group has been relatively overlooked in the context of childhood overweight^(12,13)^. Understanding the factors contributing to childhood overweight in under-five children, including various maternal and child factors, is vital for early prevention and intervention, leading to significant long-term health benefits. The NFHS, a key source of comprehensive health and nutrition data across India, is instrumental in understanding the burden of childhood overweight in the country. This analysis aims to study the prevalence and risk factors of childhood overweight at a national level, considering child, maternal and household factors. The growing prevalence of overweight-related non-communicable diseases could significantly challenge India’s healthcare infrastructure, which is already burdened with infectious diseases and undernutrition.

## Methods

The primary data for this analysis were sourced from the NFHS-5. This survey was conducted in two phases. The first phase, from 17 June 2019 to 30 January 2020, included surveys in seventeen states and five Union Territories. The second phase, from 2 January 2020 to 30 April 2021, involved surveys in eleven states and three Union Territories. It was conducted by seventeen field agencies, and information was collected from 636 699 households, 724 115 women and 101 839 men. The first tier of the sampling method involved selecting districts as the primary sampling units. Districts were stratified into urban and rural areas. In the rural areas, the primary sampling units were further stratified based on literacy rates. In urban areas, the primary sampling units were further stratified based on the percentage of Scheduled Caste and Scheduled Tribe populations. The second tier of the sampling method involved selecting households within each primary sampling units. Independent sampling probabilities were applied at each stage and cluster to determine the sampling weights. This approach ensures a representative sample and accurate results^(14)^.

For this analysis, we utilised the ‘children recode file’ (IAKR7EFL) in Stata file format available from the Demographic Health Survey website (Link: The DHS Program – India: Standard DHS, 2019–21 Dataset). This file contains comprehensive information on each child born to the interviewed women in the 5 years preceding the survey. The data included health and immunisation status, details of the mother’s pregnancy and postnatal period and socio-economic and demographic data at the household level. The variable names and coding categories have already been published along with the data. In addition, we utilised the children’s recode file of the NFHS-4 dataset to create graphical representations of the BMI Z-score distribution and to conduct temporal trend analyses, national and state-wise across surveys.

**Fig. 1.**
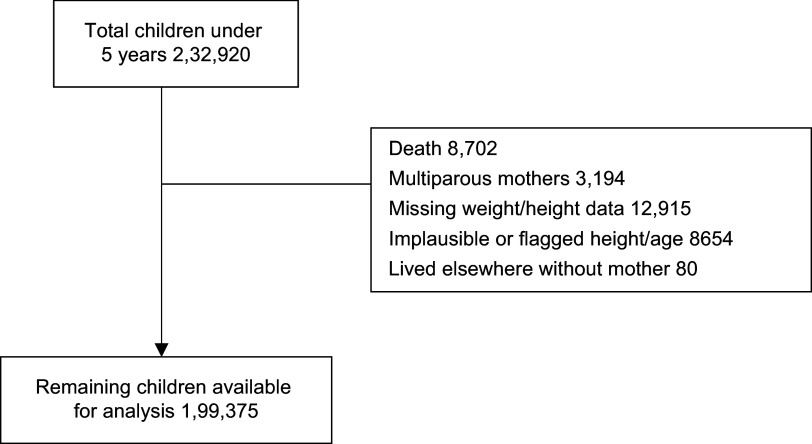
Flow diagram showing children aged 0–59 months included in the study for analyses from the NFHS-5, India.

In the dataset, 232 920 under-five children were born in the last 5 years. However, several exclusions were made to refine the dataset. The current analysis was restricted to 199 375 children aged 0–59 months for whom BMI Z-score was within plausible limits, that is, between –5 sd and +5 sd of the WHO child growth standards^(15)^, aiming to exclude obvious data entry or measurement errors, as per the WHO child growth standards. See Fig. 1 for details.

### Outcome variable

The primary outcome was overweight, defined as a BMI Z-score greater than two sd from the median, following the WHO Child Growth Standards^(15)^. This definition may include some instances of obesity typically defined as a BMI Z score more than +3 sd above the WHO growth standard median for children.

The variables analysed were grouped into child-, maternal- and household-level factors. The specific categories of these variables are given in the Supplementary file (online Supplementary Table 1). Child characteristics included age (in months), gender and birth weight. Birth weight was categorised into five groups: ≤ 1 kg, 1–1·5 kg, 1·5–2·5 kg, 2·5–3·5 kg and 3·5–4·5 kg based on observed inflection points in the distribution of birthweight with BMI Z-score, which represent overweight and non-overweight children, across the birth weight spectrum, as shown in the Supplementary file (online Supplementary Fig. 1). Other child factors considered include the season of measurement (Monsoon: June–September, Summer: March–May and Winter: October–February), birth order (1, 2, 3, 4 and 5+), mode of delivery (vaginal or caesarean section), current breast-feeding status if age less than 24 months (no or yes) and infant and young child feeding variables such as minimum dietary diversity (consuming five out of eight food groups provided during the day or night preceding the survey: breastmilk, grains, roots and tubers, legumes and nuts, dairy products, flesh foods, eggs, vitamin A rich fruits and vegetables and other fruits and vegetables) and minimum meal frequency (receiving solid or semi-solid food at least twice a day for breastfed infants 6–8 months and at least three times a day for breastfed children 9–23 months and solid or semi-solid foods from at least four food groups not including the milk or milk products food group), minimum acceptable diet (breastfed children – minimum dietary diversity and minimum meal frequency and non-breastfed children – minimum dietary diversity but excluding the dairy products category (four out of six groups) and minimum meal frequency and two or more milk feeds), stunting (height for age < −2 sd) and history of fever or diarrhoea 2 weeks preceding the survey^(16)^.

Maternal characteristics included age, education (no formal education, primary, secondary and higher), employment status, number of antenatal care visits, mass media exposure, information on receiving food supplements during the antenatal and postnatal period and maternal BMI. The BMI is divided into four categories: thin (less than 18·5 kg/m^2^), normal (18·5–24·9 kg/m^2^), overweight (25–29·9 kg/m^2^) and obesity (greater than or equal to 30 kg/m^2^). Maternal anthropometry data were only collected for mothers of children born 5 years preceding the survey. Household-level characteristics include place of residence (rural or urban), region (divided into six subdivisions: North: Punjab, Himachal Pradesh, Uttarakhand, Haryana, Chandigarh, Rajasthan, Jammu and Kashmir and Delhi; Central: Madhya Pradesh, Chhattisgarh and Uttar Pradesh; East: West Bengal, Bihar, Jharkhand and Odisha; Northeast: Nagaland, Assam, Manipur, Mizoram, Meghalaya, Tripura and Sikkim; West: Goa, Dadra and Nagar Haveli, Maharashtra, Daman and Diu and Gujarat; South: Andhra Pradesh, Karnataka, Kerala, Telangana, Tamil Nadu, Lakshadweep and Puducherry) and wealth quintile region (poorest, poorer, middle, richer and richest).

### Statistical analysis

Descriptive statistics were used to describe the distribution of the exposure and outcome variables. Categorical variables are described as proportions and 95 % CI.

We stratified the dataset into three age categories: 0–5, 6–23 and 24–59 months. We identified predictors for overweight within these age categories. We undertook these analyses and computed the weighted prevalence, adjusting for the survey’s complex design^(17)^.

We estimated the overall prevalence of overweight at both national and state level. We then compared with data from the NFHS-4 dataset to identify trends and changes over time. To identify predictors of overweight, both univariate and multiple logistic regression analyses were performed. The multiple logistic regression model was built using a stepwise approach. Variables with a *P* value of ≤ 0·2 in the univariate analysis, along with all contextually important variables, were included in the initial model. Subsequently, one variable with the highest *P* value was removed at each iteration, and the model was refitted. This iterative process continued until all variables in the model reached significance at the 0·05 level^(18)^. Variables available and utilised for building models across the age groups of 0–5, 6–23 and 24–59 months are given in the Supplementary file (online Supplementary Table 2).

Adjusted OR and their 95 % CI were calculated to describe the associations between the exposure variables and the outcome. The statistical analysis used STATA version 16·1^(19)^. To account for the complex survey design, including sampling weights, clustering and stratification, we used the *‘svy’ family of* commands which adjust the results of a statistical analysis for survey settings specified by the *svyset* command. The distribution of BMI Z-scores in NFHS-4 and NFHS-5 was depicted using Epanechnikov kernel density plots, which assign higher weights to closer data points for a smoother representation of the underlying distribution^(20)^. This visualisation helps reveal potential shifts in childhood BMI across the surveys. We compared the difference in BMI Z-scores percentiles in these surveys using the *qreqplot* command in Stata.

### Handling of missing values

Children who were not weighed and measured and whose values for weight and height were not recorded are excluded from the denominators and the numerators. In the dataset, children whose birth date is missing or unknown are automatically assigned day 15 as a default value. Children with implausible or invalid z-scores are excluded from the analyses.

## Results

The prevalence of overweight varied across different demographic and socio-economic factors (Table 1). The prevalence was highest in the youngest and lowest in the oldest age category.

**Table 1.** Prevalence of overweight (BMI Z-score >+2 sd) by socio-demographic and household characteristics of under-five children in India, as per National Family Health Survey 5 (Numbers; proportion and 95 % confidence intervals)

	*n* 199 375	Proportion	95 % CI
Overall (0–59 months)	9350	3·9	3·9, 4·13
Age in months			
0–5	1305/19 761	6·1	5·5, 6·5
6–23	3488/59 077	5·1	4·9, 5·4
24–59	4557/123 377	3·1	2·9, 3·3
Child-level factors			
Birth weight categories (kg)			
< 1·000	38/891	3·3	2·1, 5·2
1·000–1·500	91/2974	3·1	2·3, 4·1
1·501–2·500	2446/62 559	3·5	3·3, 3·7
2·501–3·500	5222/105 453	4·3	4·2, 4·5
3·501–4·500	670/10 503	5·1	4·5, 5·8
Exclusive breast-feeding (6 months)			
No	541/8306	5·9	5·2, 6·7
Yes	904/13 395	6·0	5· 5, 6·6
Stunting (height for age < −2 sd)			
No	2832/127 305	1·9	1·8, 2·0
Yes	5490/71 715	6·5	6·3, 6·8
Maternal-level factors			
Maternal education			
No formal education	1859/43 627	3·6	3·3, 3·8
Primary (≤ 5 years of schooling)	1106/26 061	3·5	3·2, 3·9
Secondary (> 5 and ≤ 10 years of schooling)	4844/1 04 911	3·9	3·7, 4·1
Higher (≥ 10 years of schooling)	1541/27 616	5·3	4·9, 5·7
Maternal employment			
Homemaker	1079/23 266	4·1	3·7, 4·5
Employed outside house	8271/178 949	3·9	3·8, 4·1
Place of residence			
Urban	2127/40 481	4·8	4·5, 5·2
Rural	7223/161 734	3·7	3·6, 3·8
Maternal size			
Undernutrition (less than 18·5 kg/m^2^)	1173/37 684	2·9	2·7, 3·1
Normal (18·5 kg/m² to 24·9 kg/m²)	6044/127 266	4·0	3·9, 4·2
Overweight (between 25 kg/m² and 29·9 kg/m²)	1556/28 260	4·6	4·3, 5·0
Obese (greater than 30 kg/m²)	577/9005	5·9	5·2, 6·6
Wealth index (region)			
Poorest	1996/49 034	3·4	3·2, 3·7
Poorer	2012/44 423	3·8	3·5, 4·1
Middle	1867/39 623	4·0	3·7, 4·3
Richer	1697/35 353	4·3	4·0, 4·6
Richest	1778/33 732	4·7	4·4, 5·1
Region			
North	1964/37 157	4·3	4·1, 4·6
Central	1914/51 407	3·4	3·2, 3·6
East	1355/30 886	4·5	4·2, 4·9
Northeast	1583/21 390	6·8	6·2, 7·4
West	847/17 780	4·7	4·2, 5·2
South	1041/24 640	4·3	3·9, 4·6
Received antenatal supplementary nutrition			
No	259/6876	3·2	2·8, 3·8
Yes	6090/138 077	3·9	3·8, 4·1
Received postnatal supplementary nutrition			
No	193/4691	3·5	2·9, 4·1
Yes	5770/131 727	3·9	3·8, 4·1

It was highest among mothers with higher education (5·3 %) and lowest among those with primary education (3·5 %). Children of employed mothers showed a slightly lower prevalence (3·9 %) than those with homemaking mothers (4·1 %). Geographically, urban areas recorded a higher prevalence (4·8 %) compared with rural areas (3·7 %). A higher prevalence of overweight in children (5·9 %) was observed when the mother was overweight or obese. The prevalence increased from the poorest (3·4 %) to the richest (4·7 %) wealth quintiles. Regionally, the northeastern region recorded the highest prevalence (6·8 %). Notably, the prevalence was significantly higher among stunted children at 6·5 % compared with non-stunted children at 1·9 %. These findings highlight disparities in overweight prevalence among various subgroups.

The graph depicting birth weight against BMI Z-score demonstrated a U-shaped distribution (see online Supplementary File Fig. 1).

**Fig. 2.**
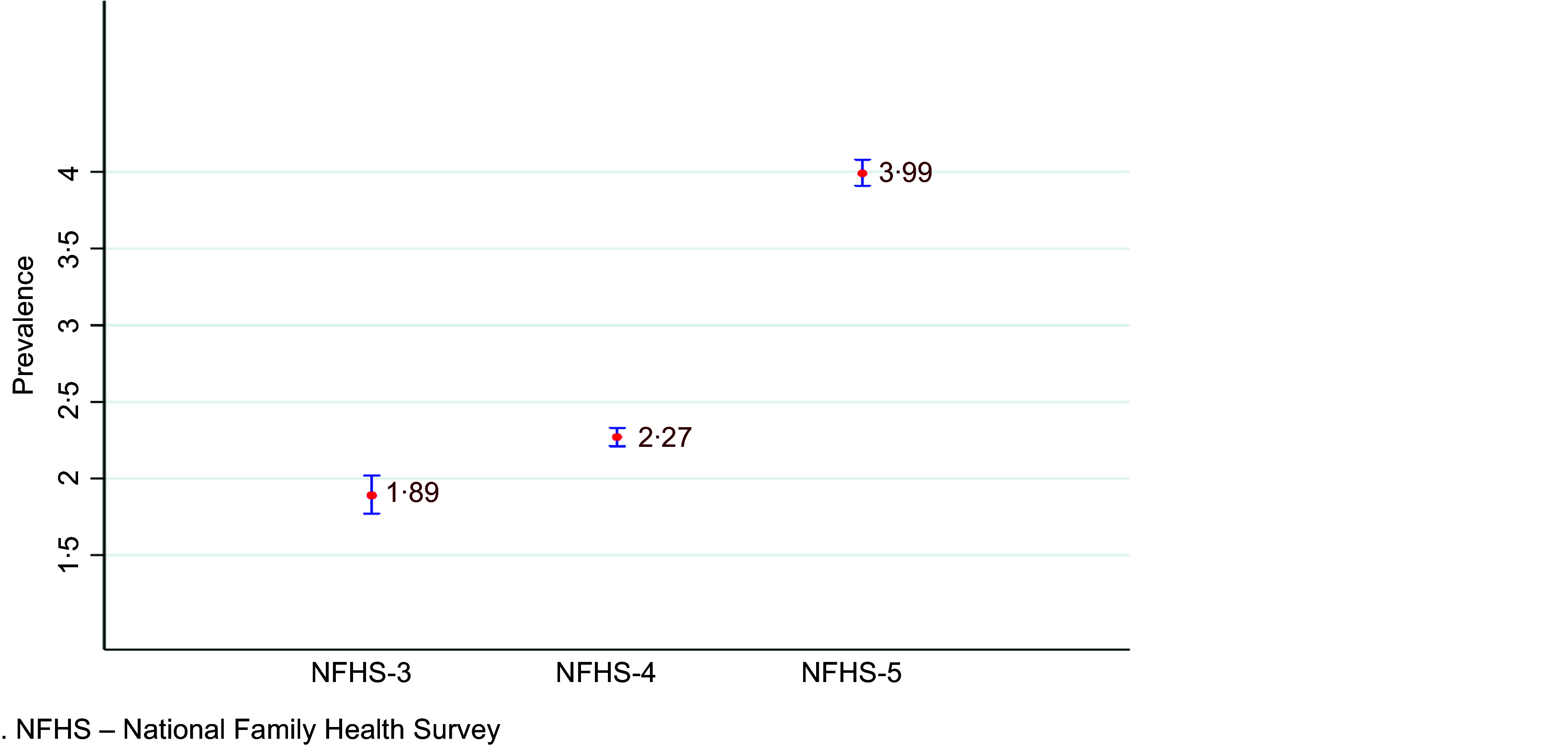
Prevalence of overweight (with 95 % confidence interval) over three surveys (NFHS-3, NFHS-4 and NFHS-5).

**Fig. 3.**
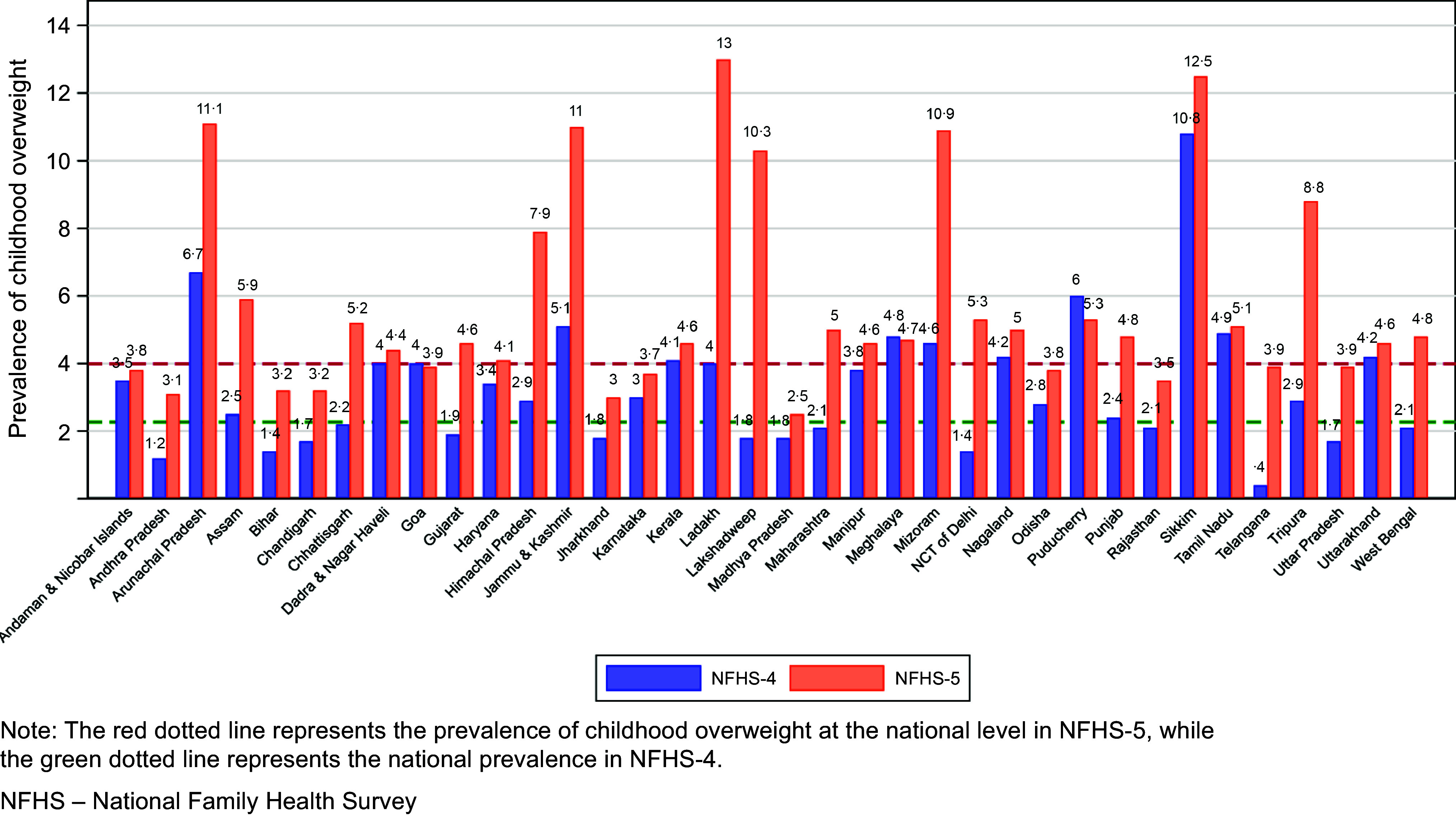
Comparison of childhood overweight prevalence by state, between NFHS-4 and NFHS-5.

Over the course of three surveys, the prevalence of overweight among children has steadily risen from 1·9 % in third round of NFHS to 2·3 % in NFHS-4 and further escalated to 4·0 % in NFHS-5, as shown in Fig. 2. This escalating trend is mirrored across the majority of states and union territories as shown in Fig. 3. Notably, several states have seen their prevalence rates nearly double or even more than double compared with the previous survey. Out of the twenty-nine states and seven union territories evaluated, twenty-four reported a prevalence of childhood overweight surpassing the national average.

**Fig. 4.**
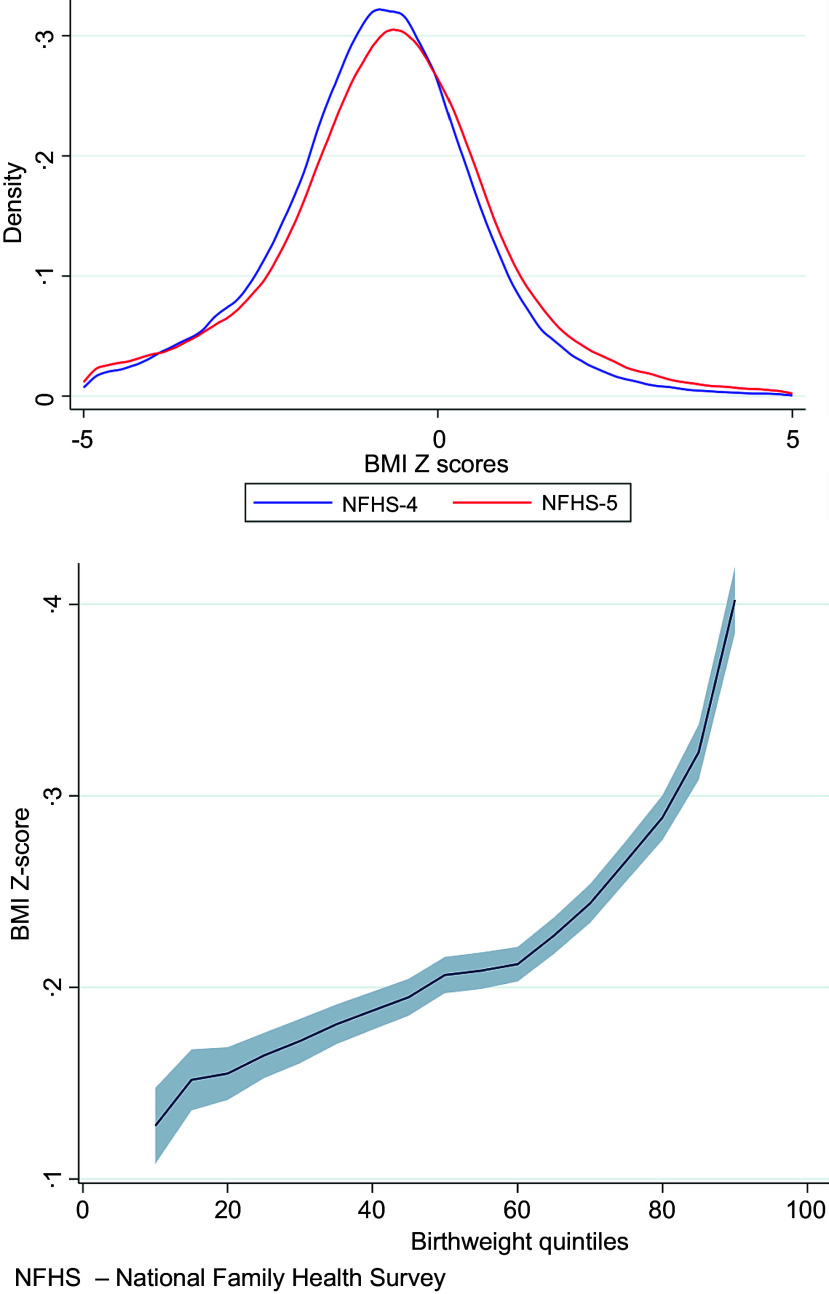
(a) Comparison of BMI Z-score distributions between NFHS-4 and NFHS-5; (b): BMI Z-scores across quintiles between NFHS-4 and NFHS-5.

Figure 4(a) shows distributions of BMI Z-scores from the NFHS-4 (blue) and NFHS-5 (red). Figure 4(b) shows the difference in BMI-Z-Scores across quintiles between the two surveys. It shows that the difference increases by increasing percentile. Specifically, the mean BMI Z-score for children under 5 years old in the NFHS-5 is higher than that in the NFHS-4.

The multiple logistic regression model revealed several significant associations between child level, maternal level and household level characteristics and the odds of children being overweight or obese (Table 2). The outcomes of the univariate regression for all characteristics at the child, maternal and household levels are detailed in the online Supplementary File (see Supplementary Tables 3, 4 and 5). Our analysis did not identify any association between exclusive breast-feeding, dietary habits and reported birth weight with childhood overweight.

**Table 2.** Factors associated with overweight (BMI Z-score >+2 sd) in the under-five age group in the model (National Family Health Survey-5) (Adjusted odds ratio and 95 % confidence intervals)

Characteristics	Categories	0–5 months		6–23 months		24–59 months	
		Adjusted† odds ratio	95 % CI	Adjusted odds ratio	95 % CI	Adjusted odds ratio	95 % CI
Season of measurement	Summer		Ref		Ref		Ref
	Winter	1·5	1·2, 1·9*	0·9	0·6, 1·3	1·6	1·3, 2*
	Monsoon	0·8	0·6, 1·5	1·3	0·9, 1·8	1·7	1·4, 1·9*
Maternal Nourishment (based on BMI)	Normal				Ref		Ref
	Underweight			0·7	0·5, 0·9*	0·7	0·6, 0·8*
	Overweight			1·1	0·8, 1·4	1·2	1·1, 1·4*
	Obese			1·3	0·8, 2·1	1·8	1·5, 2·2*
Wealth index region	Poorest		Ref		Ref		Ref
	Poorer	1·3	0·9, 1·8	1·1	0·8, 1·5	1·3	1·1, 1·5*
	Middle	1·4	0·9, 2	1·1	0·8, 1·5	1·4	1·1, 1·7*
	Richer	1·3	0·9, 1·9	1·1	0·9, 1·5	1·6	1·3, 2*
	Richest	1·0	0·7, 1·5		1·00·7, 1·4	2·1	1·7, 2·7*
Birth interval	< 24				Ref		
	24–48			1·1	0·9, 1·4		
	> 48 months			1·4	1·1, 1·7*		
Region	Northeast		Ref		Ref		Ref
	Central	0·5	0·3, 0·7*	0·4	0·3, 0·5*	0·6	0·4, 0·7*
	East	0·7	0·4, 1·03	0·5	0·4, 0·7*	0·7	0·5, 0·9*
	North	0·7	0·4, 1·01	0·5	0·4, 0·7*	0·5	0·4, 0·7*
	West	0·9	0·5, 1·6	0·7	0·5, 1·0*	0·5	0·4, 0·7*
	South	0·5	0·3, 0·8 *	0·5	0·3, 0·7*	0·5	0·4, 0·6*
Birthweight, kg	< 1·000			0·5	0·2, 1·6		
	1·000–1·500			0·5	0·2, 1·3		
	1·501–2·500				Ref		
	2·501–3·500			1·1	0·9, 1·3		
	3·501–4·500			1·7	1·2, 2·5*		
Place of residence	Urban						Ref
	Rural					0·7	0·6, 0·8*
Daily consumption of whole grains, roots and tubers	No				Ref		
	Yes			0·8	0·7, 0·9*		

* *P* < 0·05 indicates statistical significance at the 95 % confidence interval.

† Results were adjusted for the complex survey design as well as covariates in the final model.

## Discussion

The findings of this analysis highlight the growing prevalence of overweight among under-five children in India over the past decade. We also identified some associations between child level, maternal level and household level characteristics. The latest survey shows a substantial increase in prevalence at the national level compared with the previous survey, indicating a potentially serious public health problem. Most states and union territories reported prevalence rates surpassing the previous survey’s figures and the national average, necessitating urgent, targeted interventions and strategies. This concern is further magnified when considering projections from the UNICEF World Obesity Atlas, which forecasts India’s child obesity burden to reach 27 million by 2030, representing over half of the Southeast Asia region’s burden and 10 % globally. This trend of escalating the obesity burden in the entire population could drain 2·75 % of India’s Gross Domestic Product by 2060, a significant increase from the current rate of 0·8 %^(21)^.

The BMI Z-score distributions from the last two surveys show that the burden of undernutrition (i.e. those in the lower quintiles of the BMI Z score distribution) remains largely unchanged, and the burden of overweight (i.e. those in the upper percentiles) is increasing. In other words, the smallest children are not getting much larger, and the increase in mean BMI is driven by an increase among the larger children. This pattern indicates that despite ongoing efforts to mitigate malnutrition, a substantial impact on the lower quintiles is yet to be seen. In contrast, the upper quintiles are witnessing a rise in BMI, signifying an escalating burden of overweight. A similar trend has been observed in adults in India, as evidenced by a study comparing BMI Z-score distributions between third round of NFHS and 4 surveys^(22)^. This indicates that BMI distribution in children and adults may be influenced by similar factors. Therefore, interventions designed to address undernutrition and prevent overweight must consider these shared influences to ensure their effectiveness across all age groups. Policies must ensure adequate nutrition, promote healthy habits and prevent excessive weight gain. A generic approach to malnutrition is inadequate. Targeted strategies addressing specific needs at both ends of the malnutrition spectrum are imperative.

We observed significant regional differences in childhood overweight prevalence across all age groups. The Northeast region had the highest rates, while Central India had the lowest. Low economic growth and poor access to diverse food in households in the Northeast may partly explain this^(23,24)^. Diet, genetics and environment may also affect overweight risk. One potential explanation for this could be the dietary habits in these regions. For instance, the Northeast region is known for higher meat consumption, which may increase the risk of overweight^(25)^. However, it is also important to consider the economic factors at play. In many regions, individuals with lower purchasing power may have limited access to a diverse diet, often resorting to foods high in energy density and low in fibre, vitamins and minerals. This dietary characteristic is indeed associated with higher obesity prevalence^(26,27)^. Further research is needed to understand these disparities. Among the household-level factors, wealth quintile and place of residence were found to be significantly associated with childhood overweight. Maternal overweight was also found to be associated with childhood overweight, potentially due to genetic factors, shared dietary habits and lifestyle^(28)^. Between the ages of 6 and 24 months, longer birth interval was also seen to be associated with an increased risk of childhood overweight, possibly due to parents over-feeding their child with more time and resources^(29)^. A previous study that analysed data from the NFHS-4 found that child sex, age, birth weight, birth order, maternal education, number of children, age at marriage, mother’s BMI and dietary diversity score were significantly associated with childhood overweight^(30)^.

Our study found that the prevalence of overweight was highest in 0–5-month-age group. This finding challenges the common assumption that breast-feeding protects against childhood overweight. However, several factors such as genetic or hormonal influences, maternal weight gain during pregnancy and early introduction of solids or formula may also contribute to overweight risk in early infancy. Hence, it is important to consider the quality and quantity of breast milk, when assessing the association of breast-feeding with overweight. Dietary indicators, such as minimum dietary diversity, minimum meal frequency and minimum acceptable diet, are commonly used to assess the quality and quantity of complementary feeding in infants and young children. However, these indicators may not be associated with child overweight, as they do not capture the energy density, portion size or nutrient adequacy of the diets.

Stunting was not included in the logistic model building despite being an important predictor of overweight^(31,32)^. This is because both stunting and BMI are derived from the length, their associated factors will be similar and adjusting for one might bias the estimates of the others. Nevertheless, it is plausible that the overweight is linked to metabolic disturbances associated with short stature, which can result from prolonged inadequate nutritional intake, including during the prenatal phase. This process, termed metabolic programming, can modify the metabolic rate and heighten the risk of overweight due to increased body fat accumulation, aspects encapsulated in the ‘Developmental Origins of Health and Disease’ theory^(33)^. This complex relationship between stunting, overweight and metabolic programming underscores the need for further research to explore these interactions and their implications for overweight risk among children. Furthermore, there is mounting evidence suggesting that stunted children, who have a reduced potential to reach their expected height, tend to accumulate fat, resulting in increased adiposity and, consequently, a higher BMI^(34,35)^. It is important to note that the data have concurrent stunting measured at the time of measuring overweight, which might not be ideal to use as a predictive risk factor. Further research is needed to explore this relationship and its implications for overweight risk among children.

The strength of this study lies in the large representative dataset and the ability to compare different surveys. Our study also provides compelling evidence of the ‘double burden of malnutrition’ in India, underscoring the urgent need for a comprehensive approach to child health that addresses both ends of the nutritional spectrum. One of the limitations of this analysis is that it does not have information on physical activity and genetic factors that could predispose a child to overweight. These elements are crucial in understanding the complete picture of overweight and its causes. However, it is unlikely that the genetic makeup of the population has changed between the surveys. Additionally, due to the cross-sectional nature of surveys, they capture data at a specific moment, posing a challenge in observing temporal changes or determining causal relationships. We did not have data on gestational age or size at birth, which could also influence overweight risk. As a result, we were unable to categorise these infants as preterm, small for gestational age or appropriate for gestational age, rendering the birth weight variable less informative.

In conclusion, this study reveals the increasing prevalence of overweight among under-five children in India, using data from the periodic National Family Health Surveys. The increasing prevalence in early infancy particularly in certain regions, such as in the Northeast, poses significant public health concerns. While undernutrition levels have primarily remained the same, we observed a notable increase in the prevalence of overweight. Targeted interventions are urgently needed, particularly in regions with high prevalence rates.

## Supporting information

Manapurath et al. supplementary material Manapurath et al. supplementary material

## Supplementary material

For supplementary material/s referred to in this article, please visit https://doi.org/10.1017/S0007114524001582
