# Supplementary material for: Epidemiology of overweight in under-five children in India: insights from National Family Health Survey
Source: Br J Nutr. 2024 Sep 19;132(5):607–15. doi: 10.1017/S0007114524001582 (PMC11531937; doi:10.1017/S0007114524001582)
Supplement: Manapurath et al. supplementary material [file S0007114524001582sup001.docx]

Contents

[Table 1: Categories or Sub-groups of Variables Used in the Current Analysis in NFHS Dataset 2](#_Toc162142610)

[Table 2 – Variables considered for building models across the age groups of 0 to 5, 6 to 23 and 24 to 59 months. 4](#_Toc162142611)

[Figure 1: : Relationship between Birth Weight and BMI Z Score among under five children. 5](#_Toc162142612)

[Table 3: Percentage of children aged 0-59 months with Overweight (BMI-Z score >+2 SD) in India and its association with household characteristics (N=199,375;NFHS-5) 6](#_Toc162142613)

[Table 4: Percentage of children aged 0-59 months with Overweight (BMI-Z score >+2 SD) in India and its association with maternal characteristics (N=199,375; NFHS-5) 7](#_Toc162142614)

[Table 5: Percentage of children aged 0-59 months with Overweight (BMI-Z score >+2 SD) in India and its association with child characteristics (N=199,375; NFHS-5) 8](#_Toc162142615)

[Table 6: Factors Associated with Overweight (WFH Z-score >+2 SD) in the Under-Five Age Group in the model (NFHS-5 Dataset) 10](#_Toc162142616)

[Table 7: Factors Associated with Obesity (BMI Z-score >+3 SD) in the Under-Five Age Group in the model (NFHS-5 Dataset) 11](#_Toc162142617)

[Table 8: Factors Associated with Obesity (WFH Z-score >+3 SD) in the Under-Five Age Group in the model (NFHS-5 Dataset) 12](#_Toc162142618)

# Table 1: Categories or Sub-groups of Variables Used in the Current Analysis in NFHS Dataset

| **Variable Description** | **Sub-groups** |
| --- | --- |
| **Household characteristics** |  |
| Place of residence | Urban |
|  | Rural |
| Wealth index  The wealth index is a measure of socio-economic status based on possession of a number of common household items. | Richest |
|  | Rich |
|  | Middle |
|  | Poor |
|  | Poorest |
| Religion | Hindu |
|  | Muslim |
|  | Christian |
|  | Sikh |
|  | Others (Buddhist/neo-Buddhist, Jain, Jewish, Parsi/Zoroastrian, no religion |
| Caste | Scheduled caste |
|  | Scheduled tribe |
|  | Other backward class |
|  | Others (do not belong to Scheduled caste/Tribe and other backward castes) |
| Any benefit received from ICDS in last 12 months | Yes |
|  | No |
| Frequency with which food received from ICDS | 1 Almost daily |
|  | 2 At least once a week |
|  | 3 At least once a month |
|  | 4 Less often |
|  | 8 Don't know |
| **Maternal characteristics** | |
| Maternal education | No formal education |
|  | Up to Primary (≤ 5 years of schooling) |
|  | Up to Secondary (>5 and ≤10 years of schooling) |
|  | Higher Secondary and above (≥ 10 years of schooling) |
| Maternal employment status | Homemaker |
|  | Employed outside house |
| Mass media exposure | No exposure |
|  | Any exposure (frequency of watching Television or listening to radio or reading newspaper every day or in a week) |
| Maternal BMI (kg/m2) | Undernourished (less than 18.5 kg/m²) |
|  | Normal (between 18.5 kg/m² to 24.9 kg/m²) |
|  | Overweight (between 25 kg/m² and 29·9 kg/m²) |
|  | Obese (greater than 30 kg/m²) |
| Antenatal care visits | >=8 |
|  | >3 and <8 |
|  | <3 |
| **Child characteristics** | |
| Age of the child (in completed months) | 0 to 5 |
|  | 6 to 23 |
|  | 24 to 59 |
| Gender | Male |
|  | Female |
| Birth Weight Category | <1.399 kg |
|  | 1.400-1.600 kg |
|  | 1.601-4.000 kg |
|  | 4.001-4.300 kg |
|  | >4.300 kg |
| Season of measurement | Monsoon (June, July, August, September) |
|  | Summer (March, April, May) |
|  | Winter (October, November, December, January, February) |
| Birth order | 1 |
|  | 2 |
|  | 3 |
|  | 4 |
|  | ≥5 |
| Ever breastfed | Yes |
|  | No |
| Early initiation of breastfeeding | Immediately (Within 1 hour of birth) |
|  | Not immediately (After 1 hour of birth) |
| Exclusive Breastfeeding | No |
|  | Yes (Proportion of infants 0–5 months of age who received only breastmilk during the previous day) |
| Pre-lacteal feeds | No pre-lacteal feeds |
|  | Yes (in first 3 days, given anything other than breast milk) |
| Using bottle with nipple | Yes (Drank from a bottle with a nipple yesterday - under 2 years) |
|  | No |
| Diarrhea in last 2 weeks | No |
|  | Yes |
| Dietary Diversity  The proportion of children 6–23 months of age who received foods from 4 or more food groups. The 8 food groups used for tabulation of this indicator were:  a)Breastmilk  b)Grains, roots and tubers  c)Legumes and nuts  d)Dairy products (infant formula, milk, yogurt, cheese)  e)Flesh foods (meat, fish, poultry and liver/organ meats)  f)Eggs  g)Vitamin A rich fruits and vegetables  h)Other fruits and vegetables | No |
|  | Yes |
| Minimum meal frequency  a) 2 or more solid or semi-solid or soft feeds for breastfeeding children age 6-8 months, or 3 or more solid or semi-solid or soft feeds for breastfeeding children age 9-23 months; or b) 4 or more solid or semi-solid or soft or milk feeds for non-breastfeeding children age 6-23 months where at least one of the feeds must be a solid, semi-solid, or soft feed | Not Frequent |
|  | Frequent |
| Minimum acceptable diet  The proportion of children 6–23 months of age  a)Breastfed children – minimum dietary diversity and minimum meal frequency as above.  b)Non-breastfed children – minimum dietary diversity but excluding the dairy products category (4 out of 6 groups) and minimum meal frequency and 2 or more milk feeds. | No |
|  | Yes |

# Table 2 – Variables considered for building models across the age groups of 0 to 5, 6 to 23 and 24 to 59 months.

| **Variable** | **Categories** | **0-5 months** | **6-23 months** | **24-59 months** |
| --- | --- | --- | --- | --- |
| Gender | Male | **✓** | **✓** | **✓** |
|  | Female | **✓** | **✓** | **✓** |
| Birth weight |  | **✓** | **✓** | **✓** |
| Season of measurement | Winter/Pre-winter | **✓** | **✓** | **✓** |
|  | Summer | **✓** | **✓** | **✓** |
|  | Monsoon | **✓** | **✓** | **✓** |
| Birth order | 1 | **✓** | **✓** | **✓** |
|  | 2 |  |  |  |
|  | 3  4 |  |  |  |
|  | ≥5 |  |  |  |
| Ever breastfed | Yes | **✓** | **✓** | **✓** |
|  | No |  |  |  |
| Early initiation of breastfeeding | Immediately | **✓** | **✓** |  |
|  | Not immediately |  |  |  |
| Exclusive Breastfeeding | Yes | **✓** |  |  |
|  | No |  |  |  |
| Pre-lacteal feeds | Yes | **✓** | **✓** | **✓** |
|  | No |  |  |  |
| Diarrhoea in last 2 weeks | No | **✓** | **✓** | **✓** |
|  | Yes |  |  |  |
| Dietary Diversity | No |  | **✓** | **✓** |
|  | Yes |  |  |  |
| Minimum meal frequency | Not Frequent |  | **✓** | **✓** |
|  | Frequent |  |  |  |
| Minimum acceptable diet | No |  | **✓** | **✓** |
|  | Yes |  |  |  |
| Whether received Iron supplements | No | **✓** | **✓** | **✓** |
|  | Yes |  |  |  |
| Whether received Iron supplements | No | **✓** | **✓** | **✓** |
|  | Yes |  |  |  |

# Figure 1: : Relationship between Birth Weight and BMI Z Score among under five children.


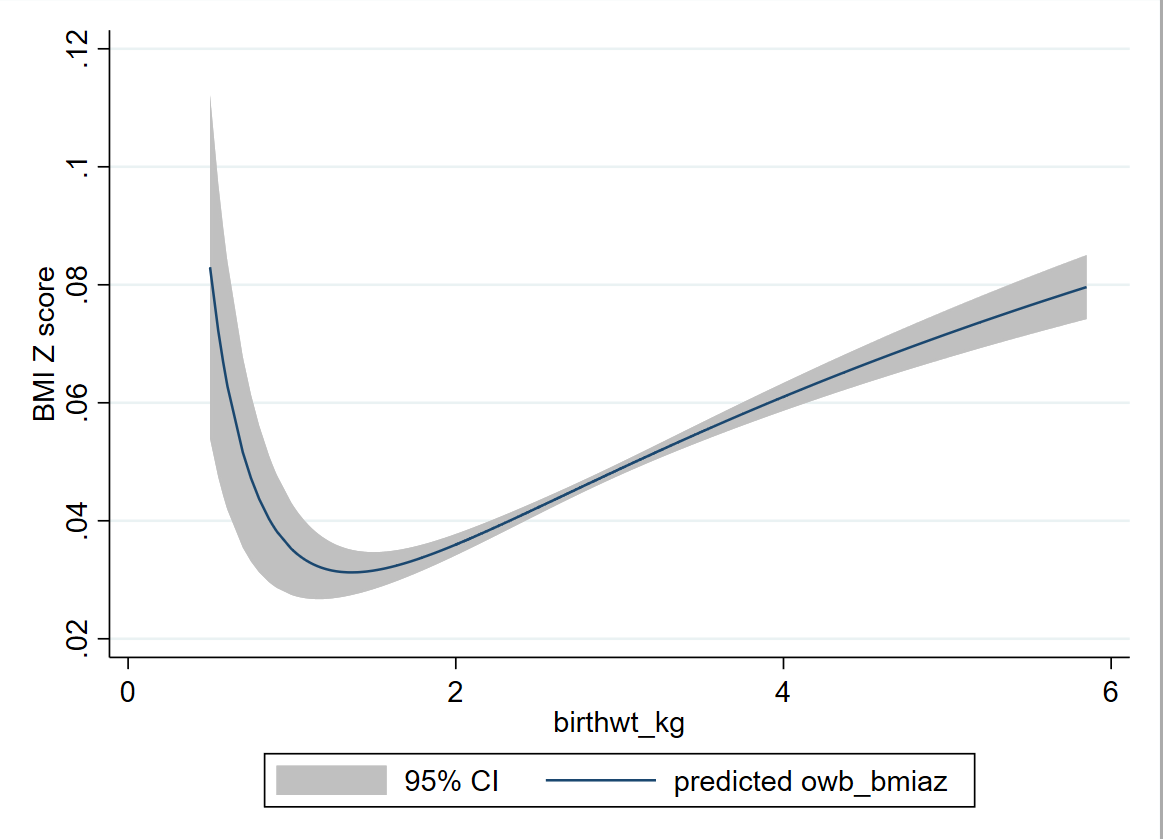


The blue line in the graph represents the predicted BMI Z score for different birth weights, calculated using a fractional polynomial model, while the shaded area around it depicts the 95% confidence interval.

# Table 3: Percentage of children aged 0-59 months with Overweight (BMI-Z score >+2 SD) in India and its association with household characteristics (N=199,375;NFHS-5)

| **Household characteristics** | **Categories** | **0-5 months** | | **6-23 months** | | **24-59 months** | |
| --- | --- | --- | --- | --- | --- | --- | --- |
|  |  | **% Overweight/**  **Obesity**  **(N=19,761)** | **Unadjusted Odds Ratio (95% CI)** | **% Overweight/**  **Obesity**  **(N=59,077)** | **Unadjusted Odds Ratio (95% CI)** | **% Overweight/**  **Obesity**  **(N=123,377)** | **Unadjusted Odds Ratio (95% CI)** |
| Place of residence | Urban | 224 (6·2) | Ref | 757 (6·5) | Ref | 1146 (4·5) | Ref |
|  | Rural | 1081 (6·7) | 0·9 [0·7 to 1·2] | 2731 (5·8) | 0·7 [0·7 to 0·9] | 3411 (3·5) | 0·7 [0·6 to 0·8] |
| Wealth index based on region | Poorest | 282 (6·0) | Ref | 704 (5) | Ref | 1010 (3·3) | Ref |
|  | Poor | 303 (7·1) | 1 [0·8 to 1·3] | 725 (5·7) | 1·2 [0·9 to 1·4] | 984 (3·6) | 1·1 [0·9 to 1·2] |
|  | Middle | 267 (7) | 1·2 [1 to 1·6] | 716 (6·2) | 1·2 [0·9 to 1·4] | 884 (3·6) | 1·2 [1 to 1·3] |
|  | Rich | 240 (6·8) | 1·2 [0·9 to 1·6] | 661 (6·2) | 1·3 [1·1 to 1·6] | 796 (3·8) | 1·2 [1·1 to 1·4] |
|  | Richest | 213 (6·2) | 1·0 [0·7 to 1·4] | 682 (6·7) | 1·5 [1·3 to 1·7] | 883 (4·4) | 1·5 [1·3 to 1·7] |
| Region | Northeast | 191 (9·5) | Ref | 622 (9·8) | Ref | 996 (4·3) | Ref |
|  | Central | 280 (4·6) | 0·5 [0·3 to 0·6] | 666 (4·2) | 0·7 [0·6 to 0·8] | 968 (3·1) | 0·6 [0·5 to 0·6] |
|  | East | 204 (6·8) | 0·7 [0·5 to 1·0] | 508 (5·5) | 1 [0·8 to 1·1] | 643 (3·4) | 0·7 [0·6 to 0·9] |
|  | North | 277 (7·1) | 0·6 [0·5 to 0·8] | 691 (5·7) | 1·8 [1·5 to 2·1] | 770 (5·7) | 0·7 [0·6 to 0·8] |
|  | West | 126 (9·4) | 1·0 [0·7 to 1·5] | 346 (6·5) | 1·1 [0·9 to 1·4] | 375 (3·4) | 0·6 [0·5 to 0·8] |
|  | South | 118 (5·1) | 0·5 [0·4 to 0·7] | 411 (5·8) | 1 [0·8 to 1·2] | 512 (3·5) | 0·7 [0·6 to 0·8] |
| Religion | Hindu | 879 (6·0) | Ref | 2339 (5·3) | Ref | 2924 (3·3) | Ref |
|  | Muslim | 208 (7·9) | 1·2 [0·9 to 1·5] | 520 (6·4) | 1·2 [1 to 1·3] | 804 (4·6) | 1·2 [1 to 1·3] |
|  | Christians | 147 (9·1) | 0·9 [0·6 to 1·3] | 414 (8·7) | 1·3 [1 to 1·7] | 521 (4·9) | 1·1 [0·9 to 1·4] |
|  | Sikh | 25 (6·6) | 1·3 [0·8 to 2·2] | 64 (6·4) | 1·4 [1 to 1·9] | 93 (4·2) | 1·2 [0·9 to 1·5] |
|  | Others | 46 (10·6) | 1·4 [0·7 to 2·7] | 151 (11·1) | 1·3 [0·8 to 2·2] | 215 (7·2) | 1·5 [1 to 2·2] |
| Caste | Scheduled caste | 256 (6·1) | Ref | 619 (5·1) | Ref | 790 (3·2) | Ref |
|  | Scheduled tribe | 336 (8·2) | 1·1 [0·9 to 1·5] | 871 (7·3) | 1·2 [0·9 to 1·4] | 1143 (4·5) | 1·1 [0·9 to 1·3] |
|  | Other backward | 434 (5·7) | 1 [0·8 to 1·2] | 1172 (5·2) | 1·2 [1·1 to 1·4] | 1424 (3·1) | 1 [0·9 to 1·2] |
|  | Others | 219 (7·5) | 1·3 [1 to 1·7] | 627 (6·7) | 1·6 [1·3 to 1·8] | 858 (4·4) | 1·5 [1·3 to 1·7] |

† OBC- Other backward classes %: Weighted percent

# Table 4: Percentage of children aged 0-59 months with Overweight (BMI-Z score >+2 SD) in India and its association with maternal characteristics (N=199,375; NFHS-5)

| **Exposure variables (Maternal characteristics)** | **Categories** | **0-5 months** | | **6-23 months** | | **24-59 months** | |
| --- | --- | --- | --- | --- | --- | --- | --- |
|  |  | **% Overweight/**  **Obesity**  **(N=19,761)** | **Unadjusted Odds Ratio (95% CI)** | **% Overweight/**  **Obesity**  **(N=59,077)** | **Unadjusted Odds Ratio (95% CI)** | **% Overweight/**  **Obesity**  **(N=123,377)** | **Unadjusted Odds Ratio (95% CI)** |
| Maternal Age | <18 years | 16(9) | Ref | 14(7·3) | Ref | 4(7·7) | Ref |
|  | 18-35 years | 1242(6·6) | 1·2 [0·6 to 2·5] | 3281(5·8) | 0·8 [0·3 to 2·1] | 4069(3·6) | 0·6 [0·2 to 2] |
|  | Above 35 years | 47(6·5) | 1 [0·4 to 2·6] | 193(7·2) | 1 [0·4 to 2·6] | 484(4·6) | 0·7 [0·2 to 2·6] |
| Maternal education | No education | 258(6·9) | Ref | 609(5·4) | Ref | 992(3·5) | Ref |
|  | Up to Primary | 148(6·4) | 0·8 [0·6 to 1·1] | 384(5·5) | 0·9 [0·8 to 1·1] | 574(3·4) | 1·1 [0·9 to 1·2] |
|  | Up to Secondary | 705(6·7) | 0·9 [0·7 to 1·2] | 1881(5·9) | 1·1 [1 to 1·3] | 2258(3·6) | 1 [0·9 to 1·2] |
|  | Higher Secondary and above | 194(6·2) | 0·8 [0·6 to 1·1] | 614(6·8) | 1·5 [1·2 to 1·7] | 733(4·7) | 1·6 [1·4 to 1·9] |
| Maternal employment status | Homemaker | 168 (6·5) | Ref | 404(5·7) | Ref | 507(3·7) | Ref |
|  | Employed outside house | 1137 (5·9) | 0·9 [0·7 to 1·1] | 3084(5·9) | 1·1 [0·9 to 1·2] | 4050(3·7) | 1 [0·8 to 1·1] |
| Mass media exposure | No exposure | 728(6·6) | Ref | 1808(5·6) | Ref | 2358(3·5) | Ref |
|  | Any exposure | 577(6·6) | 1·04 [0·9 to 1·2] | 1680(6·2) | 1·2 [1·1 to 1·3] | 2199(3·9) | 1·3 [1·1 to 1·4] |
| Maternal BMI (kg/m2) | Undernourished | 183 (5·5) | Ref | 521(4) | Ref | 469(2·2) | Ref |
|  | Normal | 912 (6·3) | 1·2 [0·9 to 1·5] | 2262(6·2) | 1·4 [1·2 to 1·7] | 2870(3·7) | 1·6 [1·4 to 1·8] |
|  | Overweight | 163 (5·5) | 1 [0·7 to 1·4] | 538(7·6) | 1·8 [1·5 to 2·1] | 855(4·6) | 2 [1·7 to 2·3] |
|  | Obese | 47 (4·5) | 0·8 [0·5 to 1·3] | 167(7·2) | 1·8 [1·4 to 2·4] | 363(6·1) | 3 [2·5 to 3·7] |
| Antenatal food supplement received | No | 46 (5·8) | Ref | 102(4·9) | Ref | 111(2·8) | Ref |
|  | Yes | 840 (5·9) | 1·01 [0·7 to 1·5] | 2355(5·7) | 1·3 [1 to 1·7] | 2895(3·5) | 1·3 [1 to 1·7] |
| Postnatal food supplement received | No | 35(6·5) | Ref | 71(5) | Ref | 87(3·2) | Ref |
|  | Yes | 754(6·4) | 0·9 [0·6 to 1·3] | 2259(5·7) | 1·3 [0·9 to 1·7] | 2757(3·4) | 1·2 [0·9 to 1·6] |

# Table 5: Percentage of children aged 0-59 months with Overweight (BMI-Z score >+2 SD) in India and its association with child characteristics (N=199,375; NFHS-5)

| **Exposure variables**  **(Child characteristics)** | **Categories** | **0-5 months** | | **6-23 months** | | **24-59 months** | |
| --- | --- | --- | --- | --- | --- | --- | --- |
|  |  | **% Overweight/**  **Obesity**  **(N=19,761)** | **Unadjusted Odds Ratio (95% CI)** | **% Overweight/**  **Obesity**  **(N=59,077)** | **Unadjusted Odds Ratio (95% CI)** | **% Overweight/**  **Obesity**  **(N=123,377)** | **Unadjusted Odds Ratio (95% CI)** |
| Gender | Female | 635 (5·6) | Ref | 1865(6·1) | Ref | 2492(3·9) | Ref |
|  | Male | 670 (6·3) | 0·9 [0·7 to 1·1] | 1623(5·7) | 0·9 [0·8 to 1] | 2065(3·5) | 0·9 [0·8 to 0·9] |
| Birthweight | <1.399 | 3 (6.6) | Ref | 17 (6.9) | Ref | 16 (4.0) | Ref |
|  | 1.400-1.600 | 3 (2.7) | 0.4 [0.1 to 3] | 14 (3.5) | 0.5 [0.2 to 1.4] | 31 (4.3) | 1.4 [0.6 to 3.2] |
|  | 1.601-4.000 | 745 (6.8) | 0.5 [0.1 to 2] | 2,104 (6.3) | 1.2 [0.6 to 2.3] | 2,466 (3.9) | 1.4 [0.7 to 2.8] |
|  | 4.001-4.300 | 7 (15.9) | 0.9 [0.1 to 7.1] | 12 (12.2) | 2.4 [0.8 to 7.2] | 9 (4.3) | 2.1 [0.6 to 7.7] |
|  | >4.300 | 6 (9.8) | 0.6 [0.1 to 3.5] | 20 (9.2) | 1.6 [0.6 to 4.1] | 26 (4.8) | 1 [0.4 to 2.7] |
| Season of measurement | Summer | 163 (4·1) | Ref | 405(3·6) | Ref | 520 (2·2) | Ref |
|  | Monsoon | 310 (5·6) | 1·3 [1·04 to 1·8] | 993 (4·8) | 1·4 [1·1 to 1·6] | 1244 (3·0) | 1·4 [1·2 to 1·6] |
|  | Winter/Pre-winter | 832 (6·8) | 1·7 [1·3 to 2·1] | 2090 (5·7) | 1·6 [1·4 to 1·9] | 2793 (3·5) | 1·6 [1·4 to 1·9] |
| Birth order | 1 | 481 (5·8) | Ref | 1532(6·6) | Ref | 1859(4) | Ref |
|  | 2 | 457 (6·6) | 1·2 [0·9 to 1·4] | 1120(5·7) | 0·8 [0·7 to 0·9] | 1469(3·6) | 0·9 [0·8 to 1] |
|  | 3 | 191 (5·2) | 0·9 [0·7 to 1·1] | 455(5·1) | 0·7 [0·6 to 0·8] | 672(3·4) | 0·8 [0·7 to 0·9] |
|  | 4 | 97 (6·2) | 1·1 [0·8 to 1·5] | 202(5) | 0·6 [0·5 to 0·7] | 301(3·3) | 0·8 [0·6 to 0·9] |
|  | ≥5 | 79 (6·3) | 1·1 [0·8 to 1·6] | 179(5·4) | 0·7 [0·5 to 0·8] | 256(3·4) | 0·9 [0·7 to 1] |
| Type of delivery | Institutional | 1142 (6·0) | Ref | 3106(5·9) | Ref | 3954 (3·2) | Ref |
|  | Home delivery | 163 (6·2) | 1·04 [0·8 to 1·4] | 382(5·7) | 0·8 [0·7 to 0·9] | 603 (2·5) | 0·8 [0·7 to 0·9] |
| Mode of delivery | Normal | 1010 (5·8) | Ref | 2638 (4·8) | Ref | 3579 (2·9) | Ref |
|  | Caesarian | 295 (6·7) | 1·2 [1 to 1·5] | 850 (6·1) | 1·3 [1·1 to 1·5] | 978 (4) | 1·4 [1·2 to 1·6] |
| Ever breastfed | No | 39 (7) | Ref | 102(6·5) | Ref | - | - |
|  | Yes | 1263 (6) | 0·8 [0·5 to 1·3] | 3209(6) | 0·9 [0·8 to 1·1] | - | - |
| Immediately breastfed | No | 701 (5·5) | Ref | 1807(5·0) | Ref |  |  |
|  | Yes | 601 (6·7) | 1·2 [1·03 to 1·5] | 1504(5·4) | 1·1 [1·0 to 1·2] |  |  |
| Exclusively breastfed | No | 450 (6·3) | Ref |  |  |  |  |
|  | Yes | 854 (5·9) | 0·9 [0·8 to 1·2] |  |  |  |  |
| Pre-lacteal feeds | No | 1107 (6·1) | Ref | 2702(5·9) | Ref |  |  |
|  | Yes | 156 (5·9) | 1 [0·7 to 1·3] | 507(6·1) | [0·9 to 1·2] |  |  |
| Dietary Diversity | No | - | - | 2629(5·8) | Ref |  |  |
|  | Yes | - | - | 859(6·1) | 1 [0·9 to 1·1] |  |  |
| Minimum meal frequency | No | - | - | 2261(5·9) | Ref |  |  |
|  | Yes | - | - | 1227(5·9) | 1 [0·9 to 1·1] |  |  |
| Minimum acceptable diet | No | - | - | 3012(5·9) | Ref |  |  |
|  | Yes | - |  | 411(6·2) | 1 [0·8 to 1·2] |  |  |

# Table 6: Factors Associated with Overweight (WFH Z-score >+2 SD) in the Under-Five Age Group in the model (NFHS-5 Dataset)

| **Characteristics** | **Categories** | **0-5 months** | **6-23 months** | **24-59 months** |
| --- | --- | --- | --- | --- |
|  |  | **Adjusted Odds Ratio (95% CI)** | **Adjusted Odds Ratio (95% CI)** | **Adjusted Odds Ratio (95% CI)** |
|  |  |  |  |  |
|  |  |  |  |  |
| Season of measurement | Summer | Ref | Ref | Ref |
|  | Winter | 1.7 [1.1 to 2.4] | 0.8 [0.3 to 2] | 1·5[1·2 to 1.9] |
|  | Monsoon | 1.0 [0.6 to 1.9] | 1.2 [0.6 to 2.6] | 1·6 [1·4 to 1·9] |
| Maternal Nourishment (based on BMI) | Normal |  | Ref | Ref |
|  | Underweight |  | 0.8 [0.5 to 1.1] | 0.7 [0.6 to 0.9] |
|  | Overweight |  | 1 [0.7 to 1.6] | 1.3 [1 to 1.6] |
|  | Obese |  | 1.1 [0.4 to 2.3] | 1.9 [1.5 to 2.5] |
| Wealth index region | Poorest | Ref | Ref | Ref |
|  | Poorer | 1 [0.7 to 1.5] | 1.1 [0.6 to 1.7] | 1.2 [0.9 to 1.6] |
|  | Middle | 1.2 [0.8 to 1.8] | 1.3 [0.8 to 2] | 1.3 [1 to 1.8] |
|  | Richer | 1 [0.7 to 1.5] | 1.3 [0.9 to 2.9] | 1.6 [1.2 to 2.3] |
|  | Richest | 0.9 [0.6 to 1.3] | 0.8 [0.5 to 1.5] | 1.9 [1.5 to 2.5] |
| Birth interval | <24 |  | Ref |  |
|  | 24 to 48 |  | 1·1 [0·8 to 1·4] |  |
|  | >48 months |  | 1·3 [1·0 to 1·7] |  |
| Region | Northeast | Ref | Ref | Ref |
|  | Central | 0.5 [0.3 to 0.7] | 0.3 [0.2 to 0.5] | 0.6 [0.4 to 0.8] |
|  | East | 0.8 [0.5 to 1.3] | 0.4 [0.3 to 0.7] | 0.7 [0.5 to 0.9] |
|  | North | 0.5 [0.4 to 0.8] | 0.5 [0.3 to 0.8] | 0.5 [0.4 to 0.7] |
|  | West | 1.0 [0.6 to 1.7] | 0.7 [0.4 to 1.2] | 0.6 [0.4 to 0.8] |
|  | South | 0.3 [0.2 to 0.5] | 0.5 [0.3 to 0.8] | 0.5 [0.4 to 0.7] |
| Birthweight, kg | <1.000 |  | 0.5 [0.1 to 2] |  |
|  | 1·000 - 1.500 |  | 0.7 [0.3 to 1.6] |  |
|  | 1·501 - 2·500 |  | Ref |  |
|  | 2.501 - 3·500 |  | 1.1 [0.3 to 4.3] |  |
|  | 3.501 - 4·500 |  | 1.6 [0.9 to 2.3] |  |
| Place of residence | Urban |  |  | Ref |
|  | Rural |  |  | 0·7 [0·6 to 0·9] |
| Daily consumption of whole grains, roots and tubers | No |  | Ref |  |
|  | Yes |  | 0.7 [0.5 to 1] |  |

Results were adjusted for the complex survey design as well as covariates in the final model.

# Table 7: Factors Associated with Obesity (BMI Z-score >+3 SD) in the Under-Five Age Group in the model (NFHS-5 Dataset)

| **Characteristics** | **Categories** | **0-5 months** | **6-23 months** | **24-59 months** |
| --- | --- | --- | --- | --- |
|  |  | **Adjusted Odds Ratio (95% CI)** | **Adjusted Odds Ratio (95% CI)** | **Adjusted Odds Ratio (95% CI)** |
|  |  |  |  |  |
|  |  |  |  |  |
| Season of measurement | Summer | Ref | Ref | Ref |
|  | Winter | 1.7 [1.3 to 2.1] | 0.8 [0.3 to 2.2] | 1.6 [1.2 to 2.2] |
|  | Monsoon | 0.9 [0.6 to 1.5] | 1 [0.4 to 2.4] | 1.5 [1.1 to 2.2] |
| Maternal Nourishment (based on BMI) | Normal |  | Ref | Ref |
|  | Underweight |  | 0.8 [0.5 to 1.2] | 0·7 [0·6 to 0·8] |
|  | Overweight |  | 0.9 [0.5 to 1.5] | 1·2 [1·1 to 1·4] |
|  | Obese |  | 1.3 [0.5 to 3] | 2.0 [1·5 to 2·2] |
| Wealth index region | Poorest | Ref | Ref | Ref |
|  | Poorer | 1.4 [0.9 to 1.9] | 1.1 [0.7 to 1.7] | 1.1 [0.9 to 1.4] |
|  | Middle | 1.3 [0.8 to 1.9] | 1.3 [0.8 to 2.2] | 1.3 [1 to 1.6] |
|  | Richer | 1.4 [0.9 to 2.0] | 1.7 [0.9 to 2.9] | 1.4 [1.1 to 1.8] |
|  | Richest | 1.0 [0.7 to 1.5] | 0.8 [0.5 to 1.5] | 1.7 [1.3 to 2.2] |
| Birth interval | <24 |  | Ref |  |
|  | 24 to 48 |  | 1·1 [0·8 to 1·4] |  |
|  | >48 months |  | 1·3 [1·0 to 1·7] |  |
| Region | Northeast | Ref | Ref | Ref |
|  | Central | 0.5 [0.3 to 0.7] | 0.3 [0.2 to 0.5] | 0·6 [0·5 to 0·7] |
|  | East | 0.8 [0.6 to 1.3] | 0.3 [0.2 to 0.5] | 0·7 [0·7 to 1.0] |
|  | North | 0.5 [0.3 to 0.8] | 0.3 [0.2 to 0.6] | 0·5 [0·4 to 0·7] |
|  | West | 1.0 [0.6 to 1.7] | 0.4 [0.2 to 0.7] | 0·6 [0·5 to 0·8] |
|  | South | 0.3 [0.2 to 0.5] | 0.4 [0.2 to 0.8] | 0·5 [0·4 to 0·7] |
| Birthweight, kg | <1.000 |  | 0·7 [0·2 to 3·6] |  |
|  | 1·000 - 1.500 |  | 0·5 [0·2 to 1·3] |  |
|  | 1·501 - 2·500 |  | Ref |  |
|  | 2.501 - 3·500 |  | 1.1 [0.9 to 1·3] |  |
|  | 3.501 - 4·500 |  | 1.7 [1.2 to 2·5] |  |
| Place of residence | Urban |  |  | Ref |
|  | Rural |  |  | 0·7 [0·6 to 0·8] |
| Daily consumption of whole grains, roots and tubers | No |  | Ref |  |
|  | Yes |  | 0.6 [0.4 to 0.8] |  |

Results were adjusted for the complex survey design as well as covariates in the final model.

# Table 8: Factors Associated with Obesity (WFH Z-score >+3 SD) in the Under-Five Age Group in the model (NFHS-5 Dataset)

| **Characteristics** | **Categories** | **0-5 months** | **6-23 months** | **24-59 months** |
| --- | --- | --- | --- | --- |
|  |  | **Adjusted Odds Ratio (95% CI)** | **Adjusted Odds Ratio (95% CI)** | **Adjusted Odds Ratio (95% CI)** |
|  |  |  |  |  |
|  |  |  |  |  |
| Season of measurement | Summer | Ref | Ref | Ref |
|  | Winter | 1.7 [1.3 to 2.1] | 0.8 [0.3 to 2.2] | 1.6 [1.2 to 2.2] |
|  | Monsoon | 0.9 [0.6 to 1.5] | 1 [0.4 to 2.4] | 1.5 [1.1 to 2.2] |
| Maternal Nourishment (based on BMI) | Normal |  | Ref | Ref |
|  | Underweight |  | 0.8 [0.5 to 1.2] | 0·7 [0·6 to 0·8] |
|  | Overweight |  | 0.9 [0.5 to 1.5] | 1·2 [1·1 to 1·4] |
|  | Obese |  | 1.3 [0.5 to 3] | 2.0 [1·5 to 2·2] |
| Wealth index region | Poorest | Ref | Ref | Ref |
|  | Poorer | 1.4 [0.9 to 1.9] | 1.1 [0.7 to 1.7] | 1·3 [1·1 to 1·5] |
|  | Middle | 1.3 [0.8 to 1.9] | 1.3 [0.8 to 2.2] | 1·4 [1·1 to 1·7] |
|  | Richer | 1.4 [0.9 to 2.0] | 1.7 [0.9 to 2.9] | 1·6 [1·3 to 2] |
|  | Richest | 1.0 [0.7 to 1.5] | 0.8 [0.5 to 1.5] | 2·1 [1·7 to 2·7] |
| Birth interval | <24 |  | Ref |  |
|  | 24 to 48 |  | 1·1 [0·8 to 1·4] |  |
|  | >48 months |  | 1·3 [1·0 to 1·7] |  |
| Region | Northeast | Ref | Ref | Ref |
|  | Central | 0.5 [0.3 to 0.7] | 0.3 [0.2 to 0.5] | 0·6 [0·5 to 0·7] |
|  | East | 0.8 [0.6 to 1.3] | 0.3 [0.2 to 0.5] | 0·8 [0·7 to 1.0] |
|  | North | 0.5 [0.3 to 0.8] | 0.3 [0.2 to 0.6] | 0·6 [0·4 to 0·7] |
|  | West | 1.0 [0.6 to 1.7] | 0.4 [0.2 to 0.7] | 0·6 [0·5 to 0·7] |
|  | South | 0.3 [0.2 to 0.5] | 0.4 [0.2 to 0.8] | 0·6 [0·5 to 0·7] |
| Birthweight, kg | <1.000 |  | 0·7 [0·2 to 3·6] |  |
|  | 1·000 - 1.500 |  | 0·5 [0·2 to 1·3] |  |
|  | 1·501 - 2·500 |  | Ref |  |
|  | 2.501 - 3·500 |  | 1.1 [0.9 to 1·3] |  |
|  | 3.501 - 4·500 |  | 1.7 [1.2 to 2·5] |  |
| Place of residence | Urban |  |  | Ref |
|  | Rural |  |  | 0·7 [0·6 to 0·8] |
| Daily consumption of whole grains, roots and tubers | No |  | Ref |  |
|  | Yes |  | 0.6 [0.4 to 0.8] |  |

Results were adjusted for the complex survey design as well as covariates in the final model.
